# Competitive non-SELEX for the selective and rapid enrichment of DNA aptamers and its use in electrochemical aptasensor

**DOI:** 10.1038/s41598-019-43187-6

**Published:** 2019-04-30

**Authors:** Ankita Kushwaha, Yuzuru Takamura, Koichi Nishigaki, Manish Biyani

**Affiliations:** 1 Department of Bioscience and Biotechnology, Japan Advanced Institute of Science and Technology, 1-1 Asahidai, Nomi City, Ishikawa, 923-1292 Japan; 2 BioSeeds Corporation, JAIST venture business laboraotry, 1-1 Asahidai, Nomi City, Ishikawa, 923-1292 Japan

**Keywords:** DNA, Screening

## Abstract

The SELEX (Systematic Evolution of Ligands by EXponential enrichment) method has been used successfully since 1990, but work is still required to obtain highly specific aptamers. Here, we present a novel approach called ‘Competitive non-SELEX’ (and termed as ‘SELCOS’ (Systematic Evolution of Ligands by COmpetitive Selection)) for readily obtaining aptamers that can discriminate between highly similar targets. This approach is based on the theoretical background presented here, in which under the co-presence of two similar targets, a specific binding type can be enriched more than a nonspecifically binding one during repetitive steps of partitioning with no PCR amplification between them. This principle was experimentally confirmed by the selection experiment for influenza virus subtype-specific DNA aptamers. Namely, the selection products (pools of DNA aptamers) obtained by SELCOS were subjected to a DEPSOR-mode electrochemical sensor, enabling the method to select subtype-specific aptamer pools. From the clonal analysis of these pools, only a few rounds of *in vitro* selection were sufficient to achieve the surprisingly rapid enrichment of a small number of aptamers with high selectivity, which could be attributed to the SELCOS principle and the given selection pressure program. The subtype-specific aptamers obtained in this manner had a high affinity (e.g., *K*_D_ = 82 pM for H1N1; 88 pM for H3N2) and negligible cross-reactivity. By making the H1N1-specific DNA aptamer a sensor unit of the DEPSOR electrochemical detector, an influenza virus subtype-specific and portable detector was readily constructed, indicating how close it is to the field application goal.

## Introduction

The rapid, precise, and selective detection of viruses is absolutely required to prevent breakouts/pandemics. This is especially true of the highly infectious influenza virus. Thus, various approaches have been explored for this purpose, including antibody engineering. Among the available methods, an *in vitro* selection termed SELEX (Systematic Evolution of Ligands by EXponential enrichment) has allowed researchers to identify a diversity of DNA/RNA aptamer molecules with potential use in virus detection. SELEX is operated using an iterative cycle of three fundamental steps, namely binding, partitioning, and amplification, and it can gradually enrich target-binding DNA/RNA molecules over the selection cycle^1–3^. Although the SELEX protocol has long been performed with success^4,5^, the difficulty involved in selecting aptamers with high specificity remains^6–8^. The current approach to this problem uses “negative selection,” which is universally applied to select aptamers that bind to a molecule of interest from a pool of non-bound molecules to a particular target of no interest (thus, they are negatively selected). This approach is widely applied, and in the case of SELEX, for example, there are reports that negative selection had the greatest positive results in selecting for cell-specific aptamers^9^. Although this approach is useful, in principle, it requires multiple rounds of negative and positive selections. The SELEX process essentially requires many rounds of selection using PCR, leading to the amplification of undesired biases^10–13^. Unfortunately, the final success ratio of SELEX-based experiments has not been high^8,14,15^ although some cases were clearly successful^16,17^. Therefore, SELEX-based technology requires some effective improvements.

Here, we propose a novel approach for obtaining selective aptamers without PCR amplification procedures, namely ‘SELCOS’ (Systemic Evolution of Ligands by COmpetitive Selection), in which *in vitro* selection is performed using a solution system containing all the positive and negative targets. In this paper, we showed the plausibility of using SELCOS on close targets of influenza virus subtypes (H1N1 and H3N2). We also introduced a DEPSOR-mode electrochemical sensing method (or Apta-DEPSOR)^18,19^ for readily evaluating the specific binding of aptamers. On the whole, a powerful approach for rapidly detecting various influenza subtypes with high sensitivity is presented here, and it addresses several theoretical considerations.

## Results

### Integration of competitive selection and electrochemical evaluation

**Figure 1:**
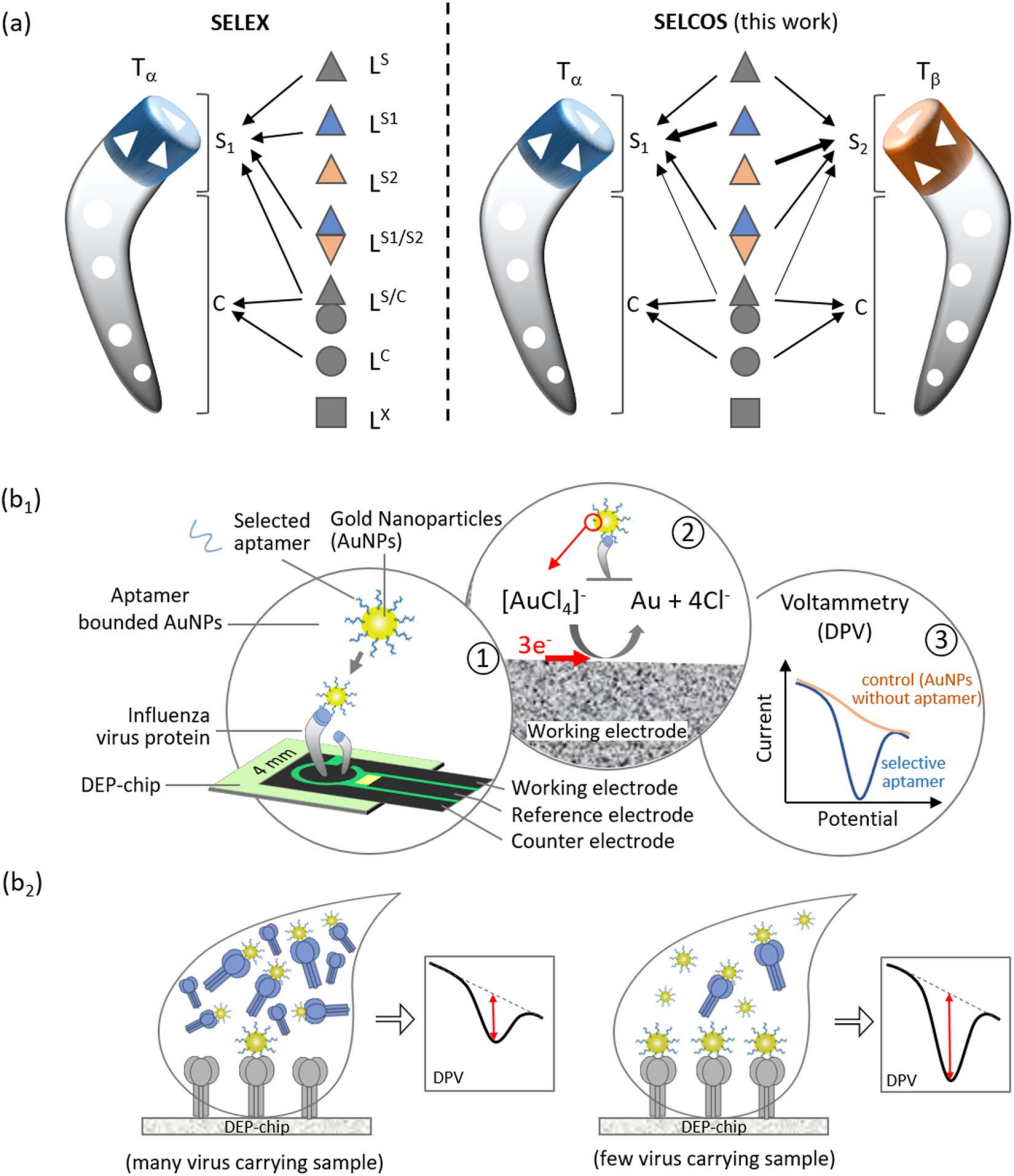
Schematic drawing of SELCOS (competitive non-SELEX) and the electrochemical sensing application. (**a**) Comparison of (conventional) SELEX and SELCOS in the ligand binding mode to the target protein. A pool of ligands is classified into 7 types in their binding mode to two different targets (T_α_ and T_β_), which are composed of the common site (C) and the specific site (S_1_ or S_2_) as follows: L^S^, L^S1^, L^S2^, L^S1/S2^, L^S/C^, L^C^, and L^X^. As shown in the figure, each ligand binds to its own binding site(s). For example, L^S^ is a ligand that can bind to the specific site of both targets (T_α_ and T_β_), while L^S1^ and L^S2^ bind to the S_1_ or S_2_ sites only, respectively. This result indicates that the same site can be recognized differently depending on a ligand. L^S1/S2^ binds to both S_1_ in T_α_ and S_2_ in T_β_. L^S/C^ binds to both site S (i.e., S_1_ and S_2_) and site C. L^C^ binds to the common site of T_α_ and T_β_. L^X^ does not bind to either T_α_ or T_β_. (**b**_**1**_) A schematic drawing of the event on the Apta-DEPSOR electrode (Aptamer-based Disposable Electrochemical Printed Sensor) in which the anti-target (influenza virus protein)-DNA aptamer-coated gold nanoparticles (AuNP) bind to the target loaded onto the working electrode of the sensor chip, followed by the electron transfer between the AuNP and the sensor surface, resulting in the generation of the DPV (differential pulse voltammetry) pattern. (**b**_**2**_) A virus protein concentration-dependent measurement of the DPV. The AuNPs are carried away with free protein when the flowing sample solution contains a large amount of virus protein. Note that the dent in the DPV curve is the signal in proportion to the bound AuNP.

**Figure 2:**
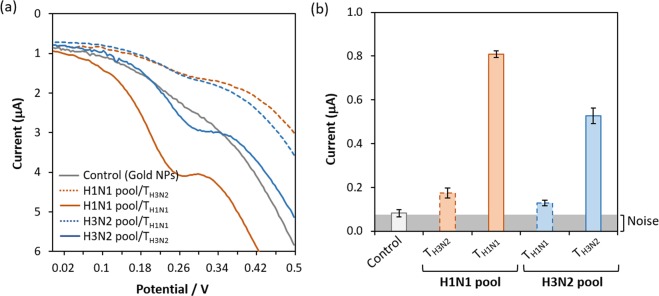
SELCOS products. Aptamer pools obtained against T_H1N1_ (i.e., target H1N1, in red) and T_H3N2_ (blue) were subjected to the electrochemical measurement using Apta-DEPSOR. (**a**) For each sample, the DPV was measured against both T_H1N1_ and T_H3N2_. (**b**) The I_pc_ (current for the signal peak) data are presented in a bar chart (using the average taken from 3 independent experiments).

As shown in Fig. 1, the pool of ligands (aptamer candidates) consists of various molecules that can be named L^S^, L^S1^, L^S2^, L^S1/S2^, L^C^, L^S/C^, and L^X^ depending on their binding nature in relation to the target molecules T_α_ and T_β_ (see details in the legend to Fig. 1). Clearly, there is a difference in their behaviors under conventional SELEX and SELCOS, which holds two or more target molecules. Those targets compete with one another for common ligands (especially, L^S^, L^S1/S2^ and L^S/C^) that can bind both targets T_α_ and T_β_ during SELCOS but exclusively T_α_ in conventional SELEX. This characteristic is the origin of the name “SELCOS”. For this reason, the ligands that bind to the S_1_ site (i.e., a T_α_-specific site) are decreased to half except L^S1^ (which binds exclusively to S_1_ site), resulting in enriched L^S1^. Clearly, this effect cannot be expected from conventional SELEX. Therefore, in the equilibrium state of the interaction between the targets and the pool of ligands, we can expect a more L^S1^-enriched (in other words, T_α_-specific ligand-enriched) result from SELCOS than SELEX. Under our experimental conditions (see the protocol in Methods and Supplementary Fig. S1), the near-saturation of binding sites with ligands is expected to be attained (an 8-fold excess of ligands against a target molecule at the final stage). The selection products (ligands) obtained in this way were processed for a negative selection (the selected ligands were treated with a mixture of all the possible targets except the genuine one and then the nonbinding ligands were collected), although this process is theoretically omittable (see Discussion). To monitor the quality of the products rapidly, we introduced a DEPSOR-mode electrochemical sensing component (Apta-DEPSOR: see Fig. 1-b_1_). Using two subtypes of influenza A virus as targets, we performed an entire SELCOS procedure and monitored the products with the Apta-DEPSOR. As in Fig. 2, the products thus obtained (and confirmed in Supplementary Fig. S2) provided the DPV response curves (Panel a) and the corresponding bar charts (Panel b) for the combination of targets (T_H1N1_ and T_H3N2_) and ligands (ligand pools against T_H1N1_ and against T_H3N2_), showing that this approach can measure the relative binding strength: the proper matching of a target and a ligand pool provided a far higher signal than those of improper matching, indicating that both SELCOS and Apta-DEPSOR are working sufficiently well. As described in Methods, the electrochemical sensing is very simple, and this integrated method is very promising for rapid and selective aptamer selection.

Note that SELCOS procedure does not depend on the PCR amplification, which is a prominent difference from conventional SELEX (see Supplementary Fig. S1) and as also discerned earlier by protocol of non-SELEX^20^. This property simplifies the whole procedure and saves experimental cost when selecting DNA aptamers. Incidentally, several studies have supported the idea that the presence of competitor molecules can enhance the specificity of the selected candidate^21–23^, though none has highlighted on the competitive effect pointed out in the work.

### Evaluation of cloned DNA aptamers

**Figure 3:**
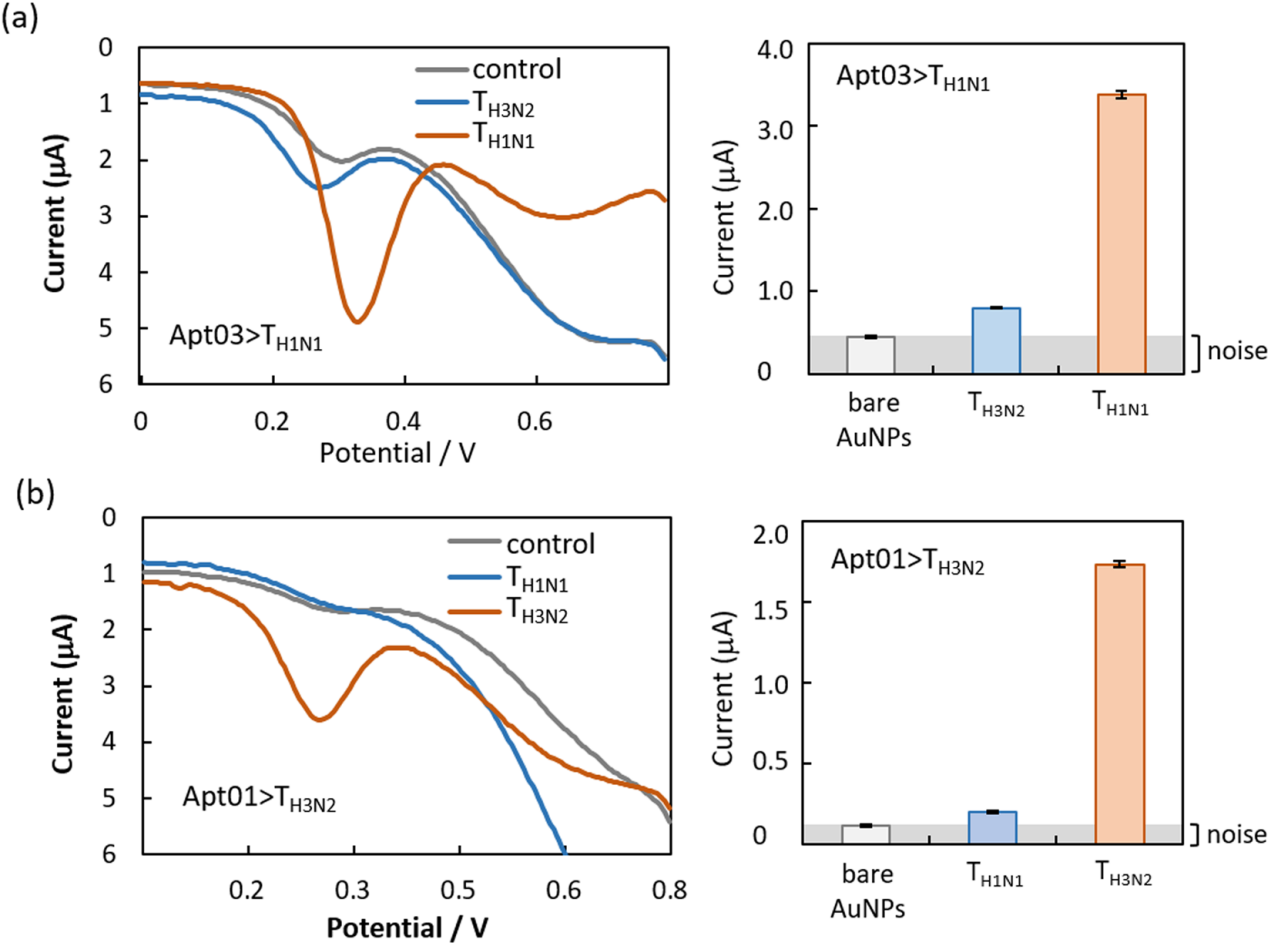
Validation of selected aptamer molecules by Apta-DEPSOR. (**a**) The aptamer, Apta03 > T_H1N1_ (namely, aptamer #03 selected against the target H1N1 protein (T_H1N1_)), was measured against T_H1N1_ and T_H3N2_. The DPV curves (left) and the corresponding bar graph (right) are shown. (**b**) The aptamer, Apt01 > T_H3N2_, was used here. “Control” (gray) indicates the signal from bare gold nanoparticles (AuNP). The concentrations of the target proteins, T_H1N1_ and T_H3N2_, were both 250 µg/mL.

**Figure 4:**
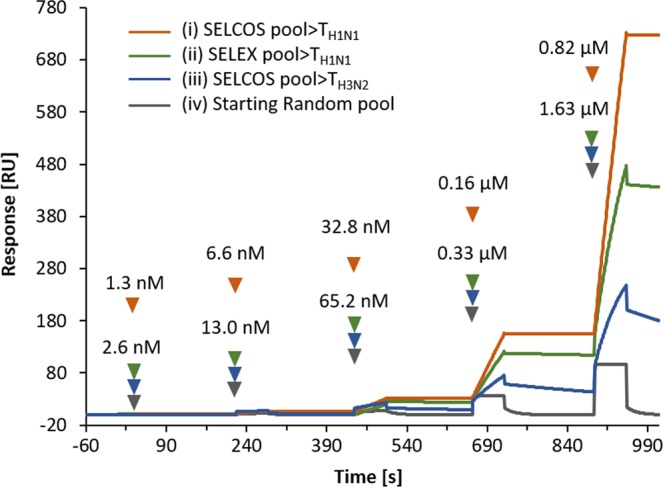
SPR analysis of the selection products with ligand T_H1N1_. Selected aptamer DNA pools were anlayzed by single-cycle kinetics SPR using a BiacoreX100. For DNA pools, a successive injections of five increasing concentrations (0.0299, 0.149, 0.746, 3.73, and 18.66 µg/mL for analyte sample (i) and 0.0592, 0.296, 1.48, 7.4, and 37 µg/mL for analyte samples (ii), (iii), and (iv) were used. The target protein binding capacity on the sensor chip surface was in levels of 2500–3000 RU (response unit). The X-axis and Y-axis represent the response (RU) and time (s) of the single-cycle kinetics sensogram, respectively. The sensograms were obtained by fitting the data using a 1:1 binding model (BioEvaluation software).

**Figure 5:**
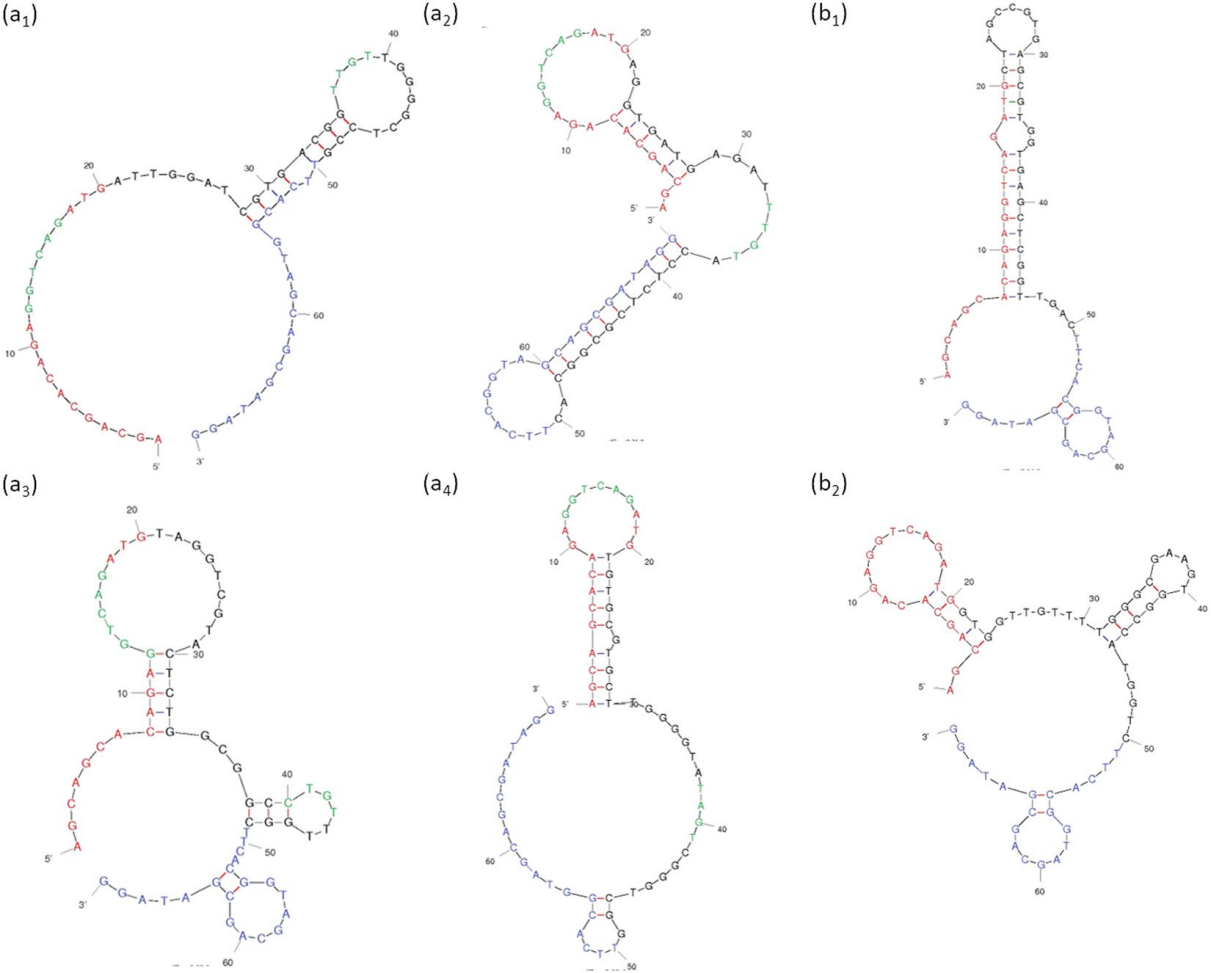
Predicted secondary structures of cloned aptamers. Some of the aptamers obtained against T_H1N1_ and T_H3N2_ were analyzed with Mfold, a secondary structure-computing program. **(a**_**1**_–**a**_**4**_**)** For the aptamers selected against T_H1N1_ (namely, Apt01 > T_H1N1_, Apt02 > T_H1N1_, Apt03 > T_H1N1_, Apt04 > T_H1N1_). **(b**_**1**_,**b**_**2**_**)** For the aptamers Apt01 > T_H3N2_ and Apto2 > T_H3N2_. Commonly appearing sequences in loop regions are highlighted for the aptamers against T_H1N1_ (incidentally, no such sequences were found in the aptamers obtained against T_H3N2_). Note that the sequence regions of 1–20 and 51–70 over the entire sequence (70 nucleotides) are primer-binding sites, and they are constant.

After SELCOS was performed with the targets T_H1N1_ and T_H3N2_, the selected pools were subjected to cloning and sequencing, providing multiple aptamers (Supplementary Fig. S3) with some of representative aptamers listed in Table 1. These aptamers were electrochemically analyzed separately as shown in Fig. 3 (with related data in Supplementary Fig. S4). The aptamer Apt01 > T_H3N2_ (denoting an aptamer named apt01 obtained in the selection targeting T_H3N2_) is shown to have an approximately 9-fold higher current signal (I_pc_, cathodic peak current) against T_H3N2_ than against T_H1N1_. Similarly, the Apt03 > T_H1N1_ aptamer is more selective for its original T_H1N1_ target (near 5-fold higher current signal) than the nontarget T_H3N2_. The binding strength to its original target was tested by SPR for these aptamers (Fig. 4 and Table 1), for which the l_pc_ values obtained from the Apta-DEPSOR method are also shown (giving a correlation score of −0.40 with the K_D_ measured by SPR). From this result, the l_pc_ value can be used to estimate the binding strength of aptamers though less exactly. The ∆G values for aptamer folding are shown in Table 1, and they present moderate stability values ranging from −6 to −12 kcal/mol with no significant correlation with the affinity K_D_ (r = −0.11). Interestingly, the secondary structures of aptamers selected against the target H1N1 (i.e., Apt01~Apt04 > T_H1N1_) have a common motif of ‘Loop1-space-Loop2’ in which common sequences are involved (GGTCAG in Loop1 and T(or C)T(or A) GT in Loop2, although the GGTCAG sequence happens to come from the primer binding site), while the aptamers selected against T_H3N2_ have no similarly remarkable characteristics as far as the evidence shows (partly shown in Fig. 5). These conserved loop regions (Loop1 and Loop2) are highly expected to interact with the target molecules^24,25^. Although it is a much simpler and more rapid method than conventional SELEX, SELCOS can attain to, sometimes, find putatively functional motif as shown here. In Table 1, it is noteworthy that the frequency score that appeared for each selection has a relatively high correlation value (r = 0.55) with the binding affinity of K_D_, conforming to a rule that ‘the higher the affinity is, the higher the population’.

**Table 1:** Properties of the aptamer DNAs selected against influenza virus proteins obtained by SELCOS.

ID for cloned aptamer^α^	Aptamer sequences^β^ (5′–3′)	Frequency of appearance (%)	ΔG^χ^ (kcal/mol)	KD^δ^ (M)	Ipc^ε^ (µA)
Apt03 > T_H1N1_	5′PBS-TAGGTCGTAC TCTGGCGGCC TGTTTGGC-3′PBS	8.33	−6.32	0.82×10^−10^	3.37 ± 0.035
Apt04 > T_H1N1_	5′PBS-TGTGCGTGCT TGGGGTATAG TCGGGTCGG-3′PBS	4.17	−5.96	0.16×10^−8^	1.23 ± 0.011
Apt02 > T_H1N1_	5′PBS-AGGTGATGAG ATTTGTACCT CTCGCGGCAC-3′PBS	8.33	−9.31	0.57×10^−7^	2.26 ± 0.011
Apt01 > T_H1N1_	5′PBS-ATTGGATCGT GACGGTTGTT GGGGCTCCG-3′PBS	12.5	−5.33	0.35×10^−4^	0.85 ± 0.036
Apt04 > T_H3N2_	5′PBS-TCTGCAGCGT GCAGGGCTGT GTGCTTACCC-3′PBS	4.17	−9.83	0.88×10^−10^	1.61 ± 0.48
Apt01 > T_H3N2_	5′PBS-CTAGCCGTGA GCGTGGTGAG CTCGGTTGAC-3′PBS	12.5	−7.51	0.14×10^−9^	1.73 ± 0.032
Apt03 > T_H3N2_	5′PBS-GCGCGGGCGG TGCGTCGGTG TCCCGCTGG-3′PBS	4.17	−12.50	0.60×10^−9^	1.11 ± 0.032
Apt02 > T_H3N2_	5′PBS-GTGGTTGTTT TGGGCGAAGT GGCCATGGTC -3′PBS	8.33	−5.51	0.17×10^−8^	1.16 ± 0.026

^α^Nomenclature for cloned aptamers were systematically assigned to be ‘serial#’ (e.g., Apt01) +‘>’ (a connector) +‘Target name’ (e.g., T_H1N1_), thus Apt01 > T_H1N1_.

^β^5′-PBS and 3′-PBS are primer binding sequences (AGCAGCACAG AGGTCAGATG and CCTATCGCTG CTACCGTGAA, respectively).

^χ^dG (kcal/mol) is free energy value and calculated from Mfold online tool.

^δ^KD (M) value is generated by BIACORE X100 using single cycle kinetics.

^ε^I_pc_ (µA) is average current value and obtained from DPV curves.

Aptamer DNAs were obtained from a single trial of SELCOS that offered two sets of aptamers, TH1N1-specific and TH3N2-specific ones. The ones listed here were chosen by the clustering analysis described in Supplementary Fig. S3. The sequence, frequency of appearance of the aptamer within a selected DNA pool, free energy for folding, dissociation constant (KD) for the binding of the target protein and aptamer, and peak current for the signal (Ipc) in the electrochemical analysis are listed.

As shown in Fig. 4, from the kinetics analysis with SPR (Biacore X100) when employing the single cycle mode analysis, data in Table 2 were obtained. Under our experimental conditions, the selected aptamer pool against T_H1N1_ in the SELCOS had a 300-fold stronger K_D_ than the value selected by conventional SELEX, and this K_D_ (1.01 × 10^−10^ M) is already close to that of the cloned aptamer Apt03 > T_H1N1_ (0.82 × 10^−10^ M). The Apt03 > T_H1N1_ aptamer is more than 100-fold stronger than that of the previously reported DNA aptamer RHA0006^26^ that was selected against influenza virus subtype H1N1. For reference, the commercial monoclonal antibody was also measured by SPR, showing the strongest affinity (2.53 × 10^−13^ M), which was surprisingly sophisticated. Interestingly, the Apt03 > T_H1N1_ aptamer exhibited a similar pattern of fitted curve, showing a high resemblance of the calculated kinetic parameters (for details, see Supplementary Fig. S5). This important data addresses some key aspects for future approaches involving the replacement of antibodies with aptamers. In any case, SELCOS provided a sufficiently competent aptamer in terms of its binding affinity.

**Table 2:** SPR analysis on the binding of selection products with the target protein used for the selection (T_H1N1_).

Ligand/Target	k_on_ (M^−1^s^−1^)	k_off_ (M^−1^s^−1^)	K_D_ (M)	R_max_ (RU)
Random ligand pool/T_H1N1_	not sufficiently bound			16
SELEX pool for T_H1N1_/T_H1N1_	6.30×10^3^	1.89×10^−4^	2.99×10^−8^	809
Compe-SELEX pool for T_H1N1_/T_H1N1_	9.34×10^3^	9.41×10^−7^	1.01×10^−10^	2651
Compe-SELEX pool for T_H3N2_/T_H1N1_	9.06×10^3^	1.80×10^−3^	1.99×10^−7^	321
T_H1N1_-Apta03/T_H1N1_	3.74×10^4^	3.08×10^−6^	0.82×10^−10^	603
Monoclonal antibody/T_H1N1_	2.33×10^5^	5.90×10^−8^	0.25×10^−12^	694
RHA0006/T_H1_ (Ref.*)	NA	NA	1.53×10^−8^	NA

‘Pool’ indicates a set of DNA aptamers that were just selected. A single cycle kinetics analysis was adopted for the SPR (surface plasmon resonance).

### Quantitation of an influenza virus subtype using Apta-DEPSOR

**Figure 6:**
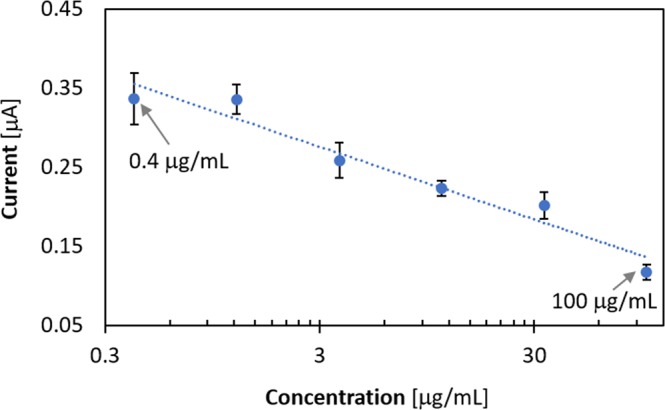
Calibration curve for measuring influenza virus A subtype H1N1 in human serum. A bound-and-free virus (protein) competition assay using Apta-DEPSOR was performed as explained in Fig. 1-b_2_. In this assay, there is competition for aptamer-coated gold nanoparticles (i.e., T_H1N1_-apt03-coated AuNP) by two phases of proteins (free and electrode surface-bound). From the regression analysis, the correlation coefficient *r* was −0.88. The lower detection limit was 0.51. The figure shows the averages taken from three trials.

An influenza subtype H1N1-specific aptamer (Apt03 > T_H1N1_)-loaded Apta-DEPSOR was fabricated as shown in Fig. 1-b_1_ and b_2_. First, the working electrode surface of a DEP chip was covered with the free target H1N1 protein (T_H1N1_), and, then, a fixed amount of AuNP (gold nanoparticles) mixed with a virus sample is introduced to the electrode, where, when there is excess AuNP relative to the free T_H1N1_ of virus sample, the excess amount of free AuNP coated with the aptamer (Apt03 > T_H1N1_) can bind to T_H1N1_ on the surface of the electrode and then be trapped and detected by the sensor. In this experimental system, the electrochemical signal will increase depending on the amount of AuNP captured by the T_H1N1_ on the electrode surface, and it will decrease proportionally depending on the amount of target T_H1N1_ protein in the sample solution. Figure 6 shows the dependence of the DEPSOR signal (current) on the concentration of applied T_H1N1_. From the resulting calibration curve, the dynamic range of the measurements ranged from 0.4 to 100 µg/mL in 5% human serum and we successfully detected T_H1N1_ in as little as 1.23 ng/L.

## Discussion

In this article, Competitive non-SELEX (SELCOS) was shown to be effective at enriching aptamers that were specific to a target protein, for which evaluations can be empowered by the introduction of the Apta-DEPSOR, a kind of electrochemical sensing device. Among the various detection devices, such as SPR, Apta-DEPSOR is useful for the current purpose due to its detectability of ligand-target interactions, readiness for operation and portability, and potential use in constructing a POC device for detecting infectious viruses with high selectivity.

The SELCOS principle is explained briefly in Fig. 1. Here, we further deepen this explanation in comparison to the conventional SELEX. The typical difference between these two methods relates to whether multiple targets can coexist or not. The current SELCOS application was operated under the experimental conditions described in Methods. There were 4 steps of imposed selection pressure (a step-wise reduction in the binding time during the increase in binding ligands with a gradual weakening from the washing effect), the successive addition of a ligand library (without PCR amplification) and one step of negative selection at the final stage (see Supplementary Fig. S1). Originally, SELEX (Systematic Evolution of Ligands by EXponential enrichment) had the following essential properties: it employs an RNA/DNA library; i) under selection pressures with increasing stringency, ii) it partitions materials into two groups that are ‘binding’ and ‘nonbinding’ (usually ‘solid phase of beads or resin’ and ‘bulk solution’), and iii) it involves the amplification of the library (usually by PCR). SELCOS has a different mode than conventional SELEX as the intermediate amplification step, i.e., PCR, of conventional SELEX is excluded in SELCOS and addition of stock supply from the original library was used between repetitive steps of partitioning. However, we must pay careful attention to the difference in the PCR amplification step of conventional SELEX and stock supply of SELCOS. First, we would like to point out that the naming of SELEX has been successful due to the appealing nature of its methodological details and the term´s nice compact ring. We have no doubt that it was a wonderful invention in the field of molecular evolution^27^. However, the words contained in “SELEX” might have been confusing since “exponential enrichment” is not correct if one believes that the molecules to be selected are enriched in an exponential mode; although the library components are amplified exponentially by PCR, no relative enrichment of a particular element occurs during this exponential amplification stage. The PCR amplification of a pool of ligands is expressed as shown below.

1 $${{\rm{L}}}_{{\rm{i}}}({\rm{t}})={{\rm{L}}}_{{\rm{i}}}({\rm{0}})\cdot {({\rm{1}}+{e}({\rm{t}}))}^{{\rm{t}}}({\rm{0}}\le {e}({\rm{t}})\le {\rm{1}})$$

2 $${{\rm{L}}}_{1}(t)/{{\rm{L}}}_{2}({\rm{t}})={{\rm{L}}}_{1}(0)\cdot {(1+e({\rm{t}}))}^{{\rm{t}}}/{{\rm{L}}}_{2}(0)\cdot {(1+e({\rm{t}}))}^{{\rm{t}}}$$

3 $$={{\rm{L}}}_{{\rm{1}}}({\rm{0}})/{{\rm{L}}}_{{\rm{2}}}({\rm{0}})={\rm{const}}{\rm{.}}$$

where L_i_(t), L_1_(t), and L_2_(t) represent the concentrations of ligands L_i_, L_1_, and L_2_ at time t, respectively, and L_i_(0), L_1_(0), and L_2_(0) are the ligand concentrations at time 0, and they are constant. The letter *e*(t) signifies the amplification efficiency at time t (the time is the integer). In PCR, ideally, *e* = 1. However, this value usually depends on the time due to the inactivation of polymerase, the decrease in primers, the increase in reactants, and so on. Since these factors are commonly influential for each template, the same *e*(t) value can be expected for each template. Thus, Eq. 3 holds true, indicating that the PCR does not change the ratio of constitutive elements before and after the PCR. In other words, no enrichment occurs during PCR, but the simple amplification of each component by the same factor (i.e., ·(1 + *e*(t))^t^) does lead to enrichment. Notably, PCR is well-known for generating alterations in the population of a library; the ratio change in the population and the mutation of DNA/RNA have no relation to the enrichment of the fitting aptamers. Parameter *e* also depends on the template DNA/RNA itself since these nucleic acids generate secondary and tertiary structures intrinsic to their sequence, and those structures are often unfavorable for the polymerization reaction (thus lessening the parameter *e* value). This effect can generate a bias in the population, but it does not denote an enrichment of the fitting aptamers; the axis of selection is quite different. Therefore, “exponential enrichment” is image-inducing wording but it is not realistic. However, since PCR, a typical process of SELEX, is not included in our method, we adopted the name “SELCOS” for our technology.

The reason (Supplementary Note) that SELCOS is superior at finding target-selective aptamers relative to conventional SELEX is discussed in the theoretical note. In brief, SELCOS can find more target-selective aptamers (L^S1^ and L^S2^ drawn in Fig. 1) due to the competitive effect of the ligand-binding between target molecules (T_α_ and T_β_). To be sure to obtain this effect, a thermodynamic equilibrium must be attained. In SELEX, successive subtraction is repeated by washing (partitioning) to enrich the aptamers of interest. During this process, due to the kinetic effect, a large population of fitting aptamers (such as L^S1^ and L^S2^) could be irreversibly lost by washing, which can be effectively circumvented by SELCOS due to the successive addition of the entire population of ligands. This approach can rationalize the experimental results in which SELCOS succeeded in yielding a higher affinity ligand pool than SELEX, as shown in Table 2 (although using a computer simulation that assumes a set of parameters might be more persuasive^28^). Surface plasmon resonance (SPR) has been extensively used to monitor binding events between analyte and ligand molecules. Thus, utilizing the similar approach for our study we compared the SPR analysis of selection pool products obtained by mode of SELCOS with the selection pool products obtained by mode of SELEX (Fig. 4). Fundamentally, we aim to compare the enrichment of the pool selected by SELCOS in presence of two targets with the pool selected by PCR-based SELEX for one target. As shown in Table 2, our observation suggested that the response value generated against ligand T_H1N1_ for initial random library was 16 RU which is negligible when compared to the SELEX pool showing 809 RU and 2.99×10^−8^ M K_D_ which exhibits enrichment to certain extent compared with initial random library. However, to our astonishment the SELCOS pool for ligand T_H1N1_ showed a relative higher response value of 2651 RU with the K_D_ 1.01×10^−10^ M. Specificity being an important aspect, we decided to check the analyte pool selected for ligand T_H3N2_ by SELCOS against ligand T_H1N1_ and observed a response of 321 RU with the K_D_ 1.99×10^−7^ M relatively lower thus indicating specificity of the selected pool by SELCOS. Henceforth, the preliminary findings from the SPR data were supportive of our theoretical understanding of SELCOS mode of action. The above-mentioned observations were determining and motivating to proceed with further analysis of candidate aptamers selected via SELCOS. At the same time, the effect decreases the relative amount of ligands (L^S/C^ and L^C^ in Fig. 1) since they bind common sites, which is multiplied when there are multiple targets, which must also contribute to the relative enrichment of target-selective aptamers. These effects are usually obtainable when the negative selection (a selection that eliminates the ligands that bind non-authentic targets) is performed. Thus, SELCOS has the ability to provide a negative selection in parallel with a positive selection. This property confers SELCOS with cost savings relative to SELEX since it can provide M-multiple different aptamers at once when M-tiple targets are adopted (although in this article, M = 2).

Clearly, in such an M-tiple target system, the ultimately selected aptamers can be expected to be exclusively selective for the relevant target with nonbinding with the other targets. Future studies about these topics are very exciting for the development of the SELCOS field.

The electrochemical sensing device introduced here (Apta-DEPSOR) for the quantitative monitoring of aptamer-target binding can generally be used for these purposes. This tool is sufficiently powerful, as shown in this study, and it has the merits of being portable and having a high cost performance (due to the disposable sensor chip used here^18^). In particular, it is favorable that its sensing part is composed of an aptamer that can be selected and evaluated by this device. Therefore, the application of this device for POC purposes (such as detecting the influenza virus at the spot of contagion) is very promising for use in the near future. Finally, we must note that developing SELCOS solely for selecting DNA aptamers is, in principle, also applicable to other selection categories such as the *in vitro* selection of peptides/proteins^29,30^ and the DNA-encoded library (DEL) selection of small molecules^31,32^. For these selections, the PCR-free nature of SELCOS is very convenient because the troublesome retagging process (such as puromycin-linker ligation to mRNA) required for those technologies, can thus be discarded (SELEX is, conveniently, free from this tagging process).

## Conclusions

The competition-driven selection of DNA aptamers using multiple targets (termed as SELCOS, Systemic Enrichment of Ligands by COmpetitive Selection) was first introduced in this study. The experimental results confirmed our success in obtaining influenza virus subtype-selective aptamers using SELCOS, which could be readily monitored with an electrochemical sensing tool (Apta-DEPSOR) as introduced here. By loading a selective aptamer as obtained (Apt03 > T_H1N1_) on its sensor unit, the feasibility of detecting the virus subtype was examined, and the detectability of subtype H1N1 ranged from 0.4–100 µg/mL. Although the situation in which the apta-DEPSOR can be useful is limited at present due to its sensitivity in the sub-µg/mL range, its portability (a merit of DEPSOR) enables us to collect important data at the POC (point of care). The theoretical consideration of SELCOS revealed its potential difference relative to conventional SELEX. In particular, its methodological advantages will be reinforced by multiple target selection, with the simultaneous acquisition of multiple aptamers of high selectivity. SELCOS, which is PCR-free, has appropriate properties for wider categories of selection such as the *in vitro* selection of peptides/proteins.

## Methods

### *In vitro* selection of DNA aptamers by competitive enrichment

Immobilization of target molecules on Ni-NTA beads: To perform SELCOS, we used the closely related subtypes of the influenza A virus H1N1 and H3N2. The targets H1N1 (abbreviated as T_H1N1_) and H3N2 (abbreviated as T_H3N2_) were immobilized onto Ni-NTA magnetic beads (20–70 µm) and Ni-NTA agarose resin beads (45–165 µm), respectively, according to the protocol for immobilizing the protein target stated by the manufacturer.

Library Design and Primers: The DNA library used for the selection was made up of a random 30-nucleotide region flanked by a 20-nucleotide primer region on both sides, specifically, 5′-AGCAGCACAGAGGTCAGATG(N30)CCTATGCGTGCTACCGTGAA-3′. For PCR amplification, the forward primer 5′-AGCAGCACAGAGGTCAGATG-3′ and the biotinylated reverse primer 5′-TTCACGGTAGCAGCGATAGG-3′ were used.

Selection Process: The plus strand ssDNA pool was heated to 90 °C for 5 min and immediately cooled to 4 °C and placed for 15 min, followed by incubation at 25 °C for 15 min. Following this step, the targets T_H1N1_ and T_H3N2_ that were immobilized on the Ni-NTA beads were incubated with 100 pmol of the ssDNA initial pool in the presence of the binding buffer (PBS buffer (pH 7.4), 100 mM NaCl, 5 mM KCl, 2 mM MgCl_2_, 1 mM CaCl_2_) for 60 min. The supernatant was then removed by washing three times with washing buffer (PBST buffer (pH 7.4 with 0.05% Tween20), 100 mM NaCl, 5 mM KCl, 2 mM MgCl_2_, 1 mM CaCl_2_). In each wash, the sample solution was briefly centrifuged at 1000 *g* for 10 s and the supernatant was removed carefully. The same procedure was repeated by 4 rounds with a successive addition of 200 pmol, 400 pmol, and 800 pmol of the ssDNA pool, changing the incubation time and washing frequency 30 min (3 times washing), 15 min (2 times washing), and 7.5 min (1 time washing), respectively. Finally, both the targets immobilized on the magnetic or nonmagnetic beads were separated by magnetic force using a magnet stand or centrifugation force (1000 *g* for 10 s), respectively, followed by the removal of the supernatant. The selected aptamer DNA pools, which are bounded on beads, were recovered by heat treatment (90 °C for 5 min followed by immediate removal of the supernatant). The selected aptamer DNA pools for T_H1N1_ and T_H3N2_ were then briefly incubated with the crude Ni-NTA beads for 15–20 min in order to remove any nonspecific candidates, if exists. The specific DNA pools selected against T_H1N1_ and T_H3N2_ were then briefly incubated with the different target solution, T_H3N2_ or T_H1N1_, to remove false positives. The specific pools for each target selected by SELCOS were amplified by PCR (initial incubation at 98 °C for 2 min, followed by 20 cycles of 98 °C for 10 s, 59 °C for 5 s, and 72 °C for 10 s, and finally, 72 °C for 4 min). Gel electrophoresis was used to monitor the successful amplifications using 8% polyacrylamide gel with 8 M urea at a temperature of 60 °C. The details of the cloning and candidate determination are mentioned in the Supporting Information section. In brief, all the selected pools were then cloned, and 20 clones were picked for sequencing. Sequence analyses were performed using the web-based tools ClustalW^33^ and Mfold^34^, for multiple sequence alignment (for details, see Supplementary Fig. S3) and secondary structure analysis, respectively.

### SPR Measurements

The SPR measurement was performed using a BIACORE X100 instrument. A sensor Chip-NTA and NTA reagent kit (GE Healthcare, Uppsala, Sweden) were used for the immobilization of the His-tag protein target for the interaction studies according to the manufacturer’s instructions. The running buffer HBS-P was used for all the experiments and 0.35 M EDTA was used for regeneration. The single-cycle mode was performed to compare the pool for ligand T_H1N1_ selected by conventional methods and SELCO. For this, 4 independent experiments were performed for the immobilization of ligand T_H1N1_ (0.01 mg/mL) in the running buffer onto the sensor surface at a level of 2500–3000 RU, with a contact time of 60 s and stabilization period of 60 s. The different analytes used for comparison were the random library, pool for T_H1N1_ selected by conventional method, pool for T_H1N1_ selected by SELCO, and pool for T_H3N2_ selected by SELCO. The selected ssDNA pool solutions of the following concentrations: 37, 7.4, 1.48, 0.296, and 0.0592 µg/mL were prepared in the running buffer and sequentially injected, starting with lowest concentration, at a flow rate of 30 µL/min for 60 s, followed by 60 s of dissociation. The kinetics of the association and dissociation were studied and compared.

To study the interaction analysis (association/dissociation) of candidate aptamers selected via SELCOS, single-cycle mode was performed for the immobilization of the ligand-target protein H1N1 with the his-tag (0.01 mg/mL) in the running buffer onto the sensor surface at a level of 2000 RU, with a contact time of 120 s and a stabilization period of 60 s. Aptamer solutions of the following concentrations: 26.66, 5.33, 1.07, 0.213, and 0.0427 µg/mL were prepared in the running buffer and sequentially injected, starting with lowest concentration, at a flow rate of 30 µL/min for 60 s, followed by 60 s of dissociation. All measurements of the binding analysis were performed in triplicate and the fitting was done for the 1:1 binding model by BiacoreX100 Evaluation software. The single-cycle mode was performed for the immobilization of the ligand protein T_H3N2_ (0.01 mg/mL) in the running buffer onto the sensor surface at a level of 1000 RU, with a contact time of 120 s and stabilization period of 60 s. Aptamer solutions of the following concentrations: 44.44, 8.89, 1.78, 0.356 and 0.0711 µg/mL were prepared in the running buffer; similar conditions for injection were used and the resulting binding curves were studied.

### Integration of SELCOS and an electrochemical sensing device (Apta-DEPSOR)

To evaluate and show the point-of-care applicability of SELCOS, we designed an electrochemical assay using our originally developed portable and disposable electrochemical printed (DEP) chip-based three-electrode sensing system DEPSOR, which functions on the principle of differential pulse voltammetry (DPV). A plot of the current relative to the voltage is generated by the electrochemical analyzer on the basis of the redox reaction^18^. As shown in Fig. 1-b_1_ and b_2_, the aptamers selected using SELCOS were integrated with DEPSOR. By performing a voltammetry assay with aptamer-conjugated AuNPs as a recognition element, a clear signal peak can be detected sensitively when a candidate aptamer binds with the target onto the working electrode of the DEP chip.

### Electrochemical Measurements

A disposable three-electrode screen-printed (DEP) chip, which was obtained from Biodevice Technology, Co. (Ishikawa, Japan), was used for this experiment. The DEP chip works on the principle of the three-electrode system for electrochemical analysis, with a carbon-based working electrode (3 mm in diameter), a counter electrode, and an Ag/AgCl reference electrode. Two µL of the recombinant proteins H1N1 and H3N2 at a concentration of 0.25 µg/µL were dropped onto the working electrode of the DEP chip, which was then incubated for one hour at 4 °C. This incubation allowed for the passive adsorption of the target protein onto the working electrode surface. After the incubation, excess target protein was rinsed three times with 100 mM PBS, and the chip was dried by gentle-blowing air. To suppress nonspecific adsorption, 3.5 µL of blocking buffer (100 mM PBS containing 1% BSA) was added to the chip; it was then incubated overnight at 4 °C. For the electrochemical analysis, the chip was further rinsed three times with 100 mM PBS buffer and dried before it could be used for the assay. A 2-µL sample made up of Au nanoparticles conjugated to the selected DNA aptamer candidates was dropped onto the target-modified DEP chip surface; the chip was then incubated for 15 min at room temperature. It was then rinsed three times with 100 mM PBS buffer and connected to an electrochemical analyzer system (Model 650 A, CH Instruments, Inc., Austin, USA). Thirty µL of 0.1 M HCl was dispensed onto the DEP chip to electrooxidate the AuNPs at a constant potential of +1.4 V for 40 s, immediately followed by DPV detection from +0.6 V to 0 V, with a step potential of 4 mV, a pulse amplitude of 50 mV, and a pulse period of 0.2 s. The selected aptamer candidates were tested for their specific subtype, and the best aptamer was selected for further analysis. For the specificity validation, the selected aptamer for T_H1N1_ was reacted with the T_H3N2_-modified DEP chip and vice-versa. Additionally, a specificity check was performed for the T_H1N1_ and T_H3N2_ pool selected by SELCOS using the same protocol. All the experiments were repeated three times to confirm the consistency of the analysis. The details of the competitive detection in human serum samples are mentioned in the Supporting Information section.

## Supplementary information

Supplementary information
