# Supplementary material for: Competitive non-SELEX for the selective and rapid enrichment of DNA aptamers and its use in electrochemical aptasensor
Source: Sci Rep. 2019 Apr 30;9:6642. doi: 10.1038/s41598-019-43187-6 (PMC6491428; doi:10.1038/s41598-019-43187-6)
Supplement: Supplementary file 1 — Supplementary information [file 41598_2019_43187_MOESM1_ESM.pdf]

Supplementary Information

Competitive non-SELEX for the selective and rapid enrichment of DNA aptamers and its use in electrochemical aptasensor

Ankita Kushwaha[a], Yuzuru Takamura[a], Koichi Nishigaki[a,b], Manish Biyani[a,b,*]

[a]Department of Bioscience and Biotechnology, Japan Advanced Institute of Science and Technology, 1-1 Asahidai, Nomi City, Ishikawa 923-1292, Japan

[b]BioSeeds Corporation, JAIST venture business laboraotry, 1-1 Asahidai, Nomi City, Ishikawa 923-1292, Japan

*Corresponding author:

Manish Biyani, PhD

E-mail: biyani@jaist.ac.jp, Tel.: +81-761-51-1665, Fax: +81-761-51-1665

Supplementary Figure S1: Schematic drawing of SELCOS (or Competitive non-SELEX). The experimental scheme adopted is shown here: 4 successive elution steps that gradually increased the concentration of the library components (1-fold to 8-fold) at the same time, gradually decreasing the washing rounds. Both of these operations favor binding whereas a concomitant decrease in the binding time is unfavorable for binding. The experimental details are described in Methods. In this figure, the competition for the 4 targets is illustrated (a 2-target competition was used in the experiment) since a multiplex type is theoretically more general. After 4 steps of partitioning without PCR amplification, a negative selection (or counter selection) is performed at the final stage.

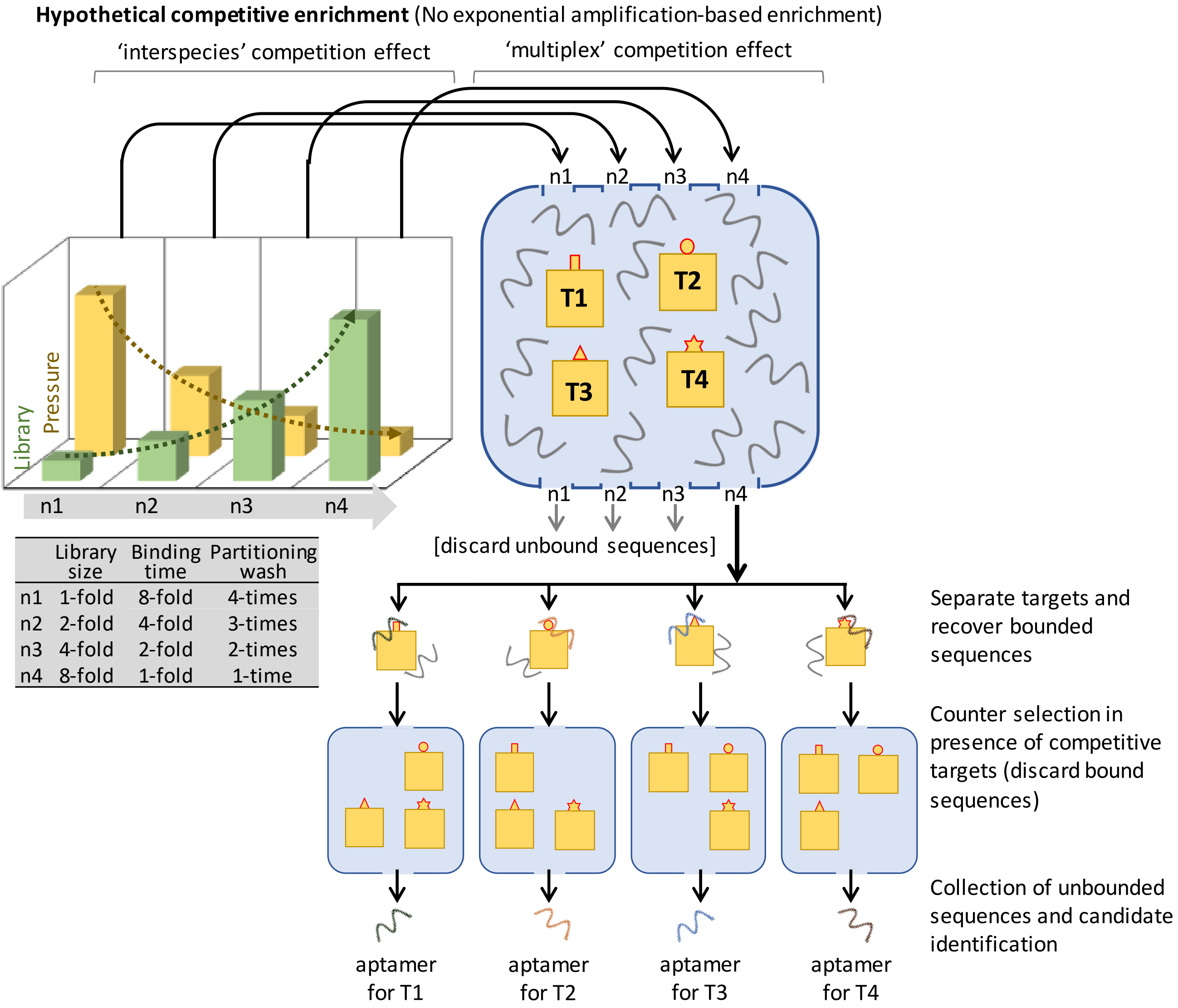

Supplementary Figure S2: Confirmation of SELCOS products by gel electrophoresis. Electrophoresis was performed under denaturing conditions (8% polyacrylamide gel, 8 M urea at 60 ℃). Obviously, the experimental products with no PCR can result in the same-sized products after selection.

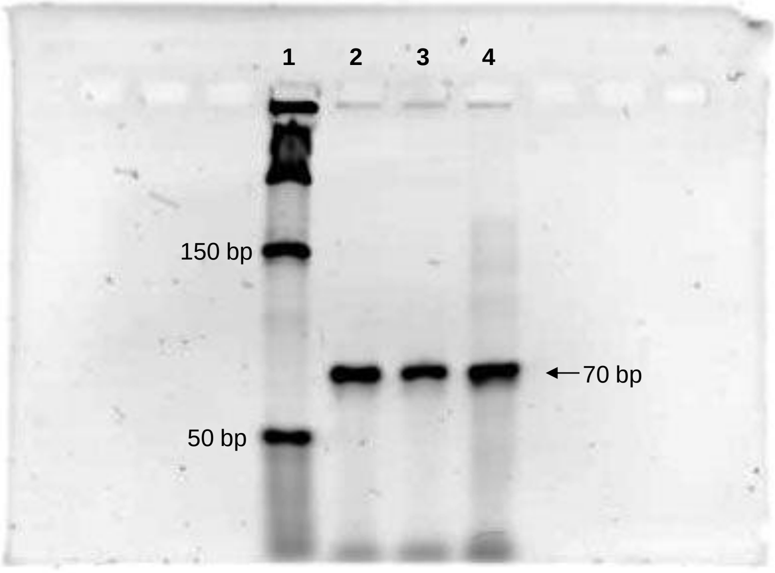

Lane 1: DNA Marker
Lane 2: Selected pool for T_{H1N1}
Lane 3: Selected pool for T_{H3N2}
Lane 4: Reference DNA

Supplementary Figure S3: **Cladograms generated for aptamer sequences** obtained by Compe-SELEX targeting H1N1 and H3N2 proteins. The encircled aptamers in these cladograms are renamed (parenthesis) and processed for further analysis (Ref.: for Clustal W-based cladogram analysis: Thompson et al., *Nucleic Acids Res*. 1994, 22, 4673. Clustal W: improving the sensitivity of progressive multiple sequence alignment through sequence weighting, position-specific gap penalties and weight matrix choice).

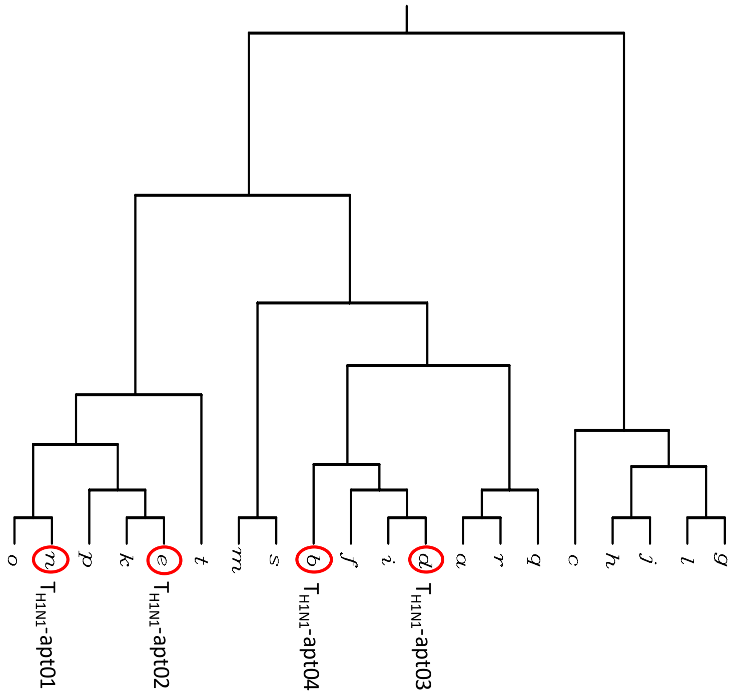

(a) Cladogram for sequences selected for T_{H1N1}

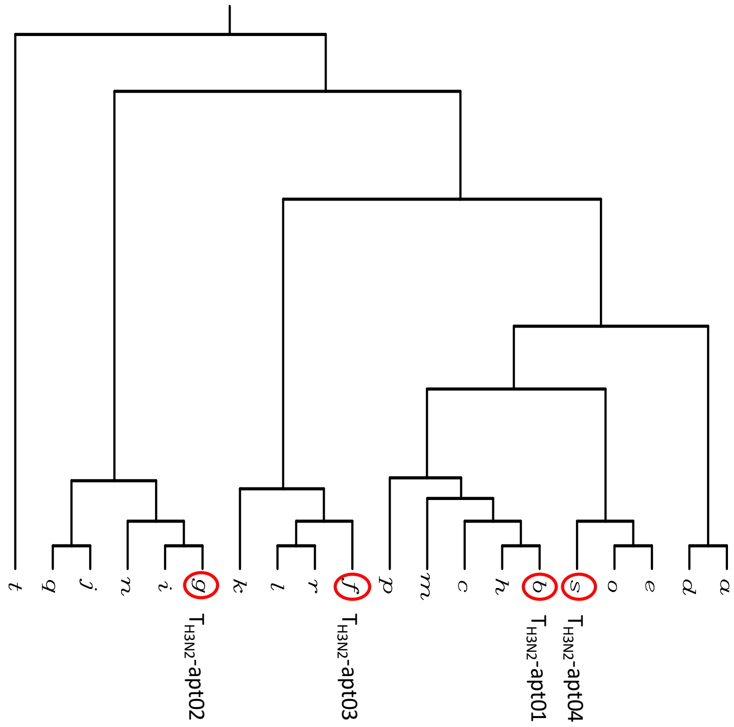

(b) Cladogram for sequences selected for T_{H3N2}

Supplementary Figure S4: Measuring the binding affinities of cloned aptamers. The DNA aptamers selected by SELCOS were subjected to Apta-DEPSOR analysis using the corresponding targets, providing the DPV curves (a and c) and its processed data (b and d). The aptamers Apt01~Apt04>T_{H1N1} were measured against the target H1N1 protein (T_{H1N1}) (a and b) and the aptamers Apt01~Apt04>T_{H3N2} against T_{H3N2} protein (c and d). In b and d, the average data for three trials are plotted.

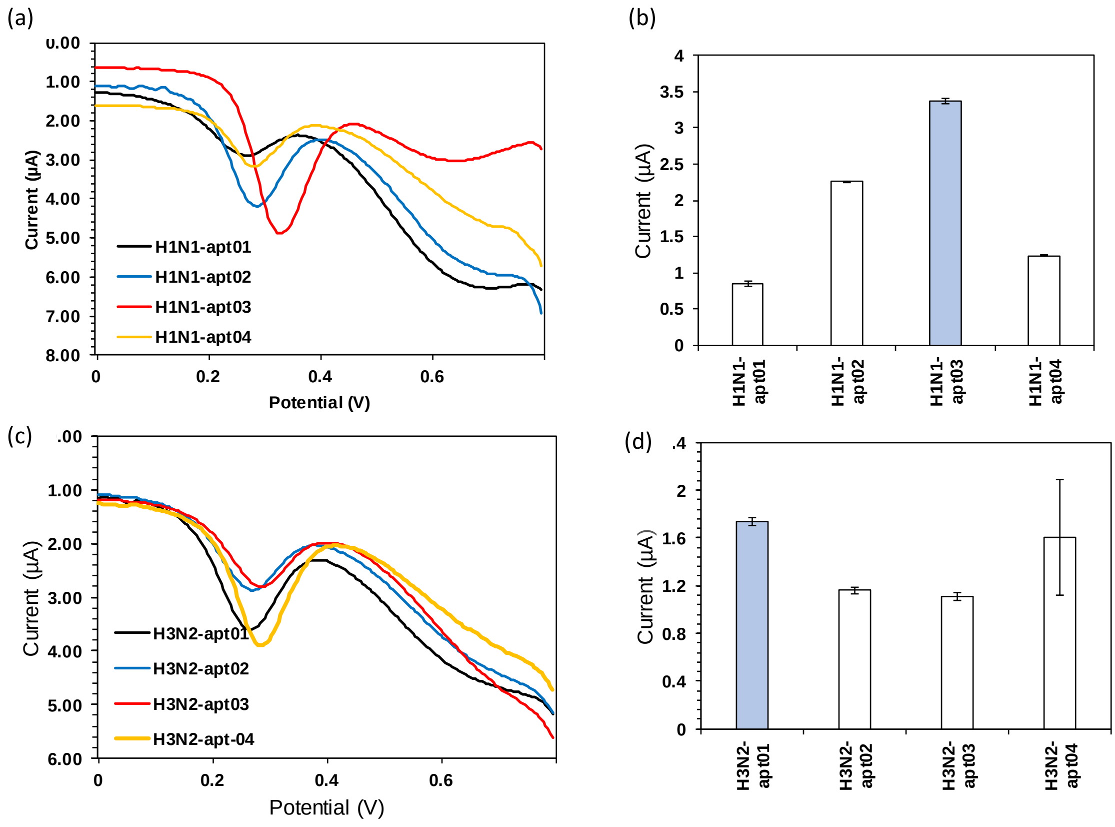

Supplementary Figure S5: SPR analysis of selected aptamer vs. antibody. Comparison of selected aptamer Apt03>T_{H1N1} by SELCOS (blue) with monoclonal antibody (orange) for target T_{H1N1} showing comparable kinetics. Single-cycle kinetics conditions for the study of interaction analysis used are as follows: Immobilization of ligand-target protein T_{H1N1} on the sensor surface to level 2000 RU, Analyte (Apt03>T_{H1N1} and mAb) solutions were prepared in the order 26.66, 5.33, 1.07, 0.213 and 0.0427 µg/ml and sequentially injected starting with lowest concentration at a flow rate of 30 µl/min and the fitting was done by 1:1 binding model by BiacoreX100 Evaluation software.

Ligand	k_{on} ($M^{-1}s^{-1}$)	k_{off} ($M^{-1}s^{-1}$)	K_D (M)
mAb	2.331×10^{5}	5.903×10^{-8}	0.253×10^{-12}
apta03	3.736×10^{4}	3.075×10^{-6}	82.34×10^{-12}

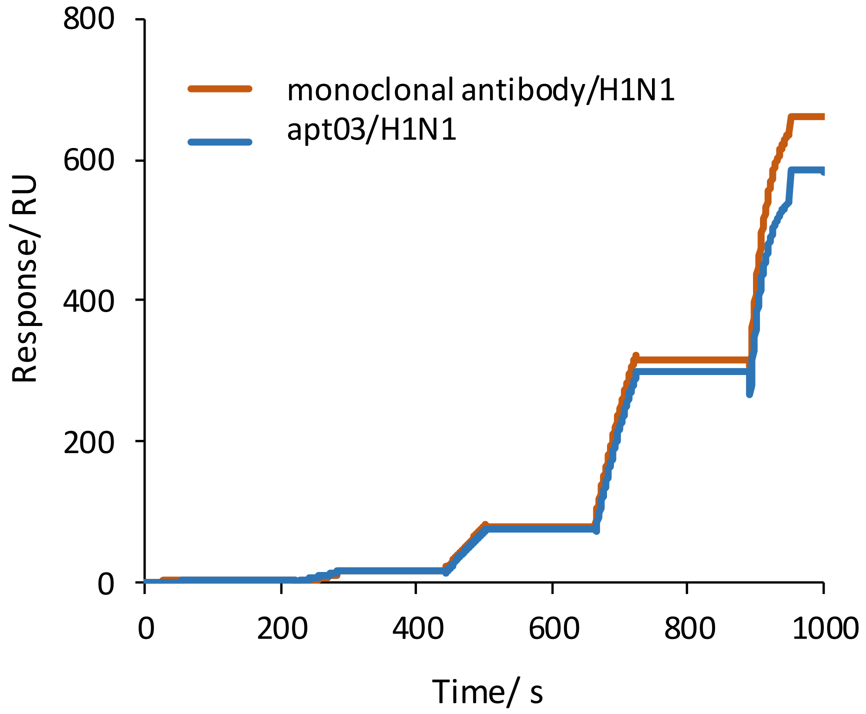

Supplementary theoretical note:

To explain the phenomenon that occurs in the competitive non-SELEX selection (SELCOS), a pool of ligands can be categorized into 7 types from the perspective of their binding to one or multiple target molecules: L^S, L^{S1}, L^{S2}, $L^{S1/S2}$, L^C, $L^{S/C}$ and L^X. Each symbol represents a ligand that binds to common site C of two targets of T_α and T_β (L^C), a ligand binding to specific site S_1 within T_α (L^{S1}), a ligand binding to specific site S_2 within T_β (L^{S2}), ligands binding to both sites S_1 and S_2 simultaneously (L^S and $L^{S1/2}$), a ligand that binds to both targets T_α and T_β ($L^{S/C}$) and the other ligands, which do not bind to either T_α or T_β (L^X). This setup can be represented by the following equations based on conservation law:

$$[L^{S1}\cdot S_1]+[L^{S1/S2}\cdot S_1]+S_1 = [S_1]_0 \quad (1)$$

$$[L^{S2}\cdot S2]+[L^{S1/S2}\cdot S_2]+S_2 = [S_2]_0 \quad (2)$$

$$[L^C\cdot C]+C = [C]_0 \quad (3)$$

$$[L^{S1}\cdot S_1]+[L^{S1}] = [L^{S1}]_0 \quad (4)$$

$$[L^{S2}\cdot S_2]+[L^{S2}]= [L^{S2}]_0 \quad (5)$$

$$[L^C\cdot C]+[L^C]= [L^C]_0 \quad (6)$$

$$[L^X] = [L^X]_0 \quad (7)$$

$$[L^{S1/S2}\cdot S_1]+[L^{S1/S2}\cdot S_2]+[L^{S1/S2}] = [L^{S1/S2}]_0 \quad (8)$$

$$[L^C]+ [L^{S1}]+[L^{S2}]+ [L^{S1/S2}]+ [L^X]+ [L^C\cdot C] + [S_1]_0 + [S_2]_0 = \Sigma L = [L_0] \quad (9)$$

where $[S_1]_0$ and $[S_2]_0$ designate the initial concentrations of sites S_1 and S_2, which are equal to $[T_\alpha]_0$ (that is, the initial concentration of the target T_α) and $[T_\beta]_0$ (that of the target T_β), respectively, and L_0 stands for the initial ensemble concentrations of ligand L. Similarly, C and $[C]_0$ represent the concentration of the common binding site and its initial one, and thus,

$$[C]_0 =[T_\alpha]_0 +[T_\beta]_0 \quad (10)$$

For convenience, we can set $[T_\alpha]_0 =[T_\beta]_0 =T_0$ operationally. Then,

$$[C]_0 = 2\cdot T_0 \quad (11)$$

This result indicates that when the initial concentration of L^C is the same under SELCOS and conventional positive SELEX, the concentration of $L^C\cdot T_\alpha$ for Competitive non-SELEX becomes half of that for the conventional form due to the following equation.

$$L^C\cdot C = L^C\cdot(T_\alpha + T_\beta) = 2L^C\cdot T_\alpha \quad (12)$$

Since L^C is a nonspecific binder (aptamer), the SELCOS can thus reduce the nonspecific aptamers through a 'nonspecific target multiplying' effect. This effect can be further

reinforced by multiplying the diversity of competitors in SELCOS as in the case of influenza subclass viruses.

Another effect of SELCOS for selecting more specific aptamers than the conventional method (typically, a combination of negative selection and positive selection) is shown in T_α) can be determined from the following Eqs. 13, 14, and 15 as derived from Eqs. 1, 2, and 8:

$$[L^{S1/S2}\cdot S_1] = [S_1]_0 - [L^{S1}\cdot S_1] - S_1 \quad (13)$$

$$[L^{S1/S2}\cdot S_2] = [S_2]_0 - [L^{S2}\cdot S_2] - S_2 \quad (14)$$

$$[L^{S1/S2}\cdot S_1] = [L^{S1/S2}]_0 - [L^{S1/S2}\cdot S_2] - [L^{S1/S2}] \quad (15)$$

Clearly, in the case of noncompetitive SELEX, the terms related to S2 located in T_β (not applied) do not appear. Now, Eq. 15 is converted to

$$[L^{S1/S2}\cdot S_1] = [L^{S1/S2}]_0 - [L^{S1/S2}] \quad (16)$$

The left-hand side is evidently larger by $L^{S1/S2\cdot S2}$ than that of SELCOS, meaning that SELCOS can decrease the amount of nonspecific aptamer ($L^{S1/S2}$) by this effect. Even so, Eqs. 13 and 14 are noteworthy. Eq. 13 represents a typical competition between different ligands competing for the same site while Eq. 14 represents latent competitor L^{S2} (i.e., how strong it is in struggling for site S_2), which has an indirect influence on the recovered amount of target aptamer L^{S1}.

The above estimation must be carefully applied under the premise of dealing with nonextreme conditions (i.e., excluding high excess concentrations of the targets ($T_0 \gg L_0$) or ligands ($T_0 \ll L_0$) so that the competitions of interest are working well). A more quantitative and parametrical approach, although not presented in this article, will be possible, as in the previous work.
